# Lifestyle consequences for rescue workers in public health emergencies: a cross-sectional study from china

**DOI:** 10.3389/fpubh.2026.1758053

**Published:** 2026-02-27

**Authors:** Qiao Chen, Yujian Song, Zhiguang Shi, Yuhan Bao

**Affiliations:** 1 The Third Medical Center, Chinese PLA General Hospital, Beijing, China; 2 Chinese People’s Armed Police Force China Coast Guard Hospital, Jiaxing, China; 3 Army Hospital 93735, Tianjin, China

**Keywords:** biosecurity, lifestyle changes, occupational health, public health emergency, rescue workers

## Abstract

**Objective:**

This study aimed to comprehensively evaluate changes in lifestyle behaviors and mental health status among rescue workers before and after a major public health emergency, and to identify key modifiable risk factors associated with psychological distress in this critical population.

**Methods:**

A cross-sectional online survey was conducted between February 23 and March 9, 2020, among rescue workers from a designated unit in China. Using a retrospective design, 1,052 valid responses were collected. Participants reported lifestyle behaviors (smoking, alcohol use, mobile phone use, physical activity, work hours, sleep quality) for both pre- and post-outbreak periods. Mental health was assessed using the PHQ-9 and GAD-7 scales. Statistical analyses included paired comparisons, correlation analysis, stratification by living status and age group, and multivariable regression models.

**Results:**

Significant adverse lifestyle changes occurred post-outbreak: increases in smoking (13.98% of smokers), alcohol consumption (6.02% of drinkers), mobile phone use (median rise from 2 to 3 h/day), and nocturnal awakenings, alongside declines in physical activity (inactivity rose from 6.56 to 17.68%) and work hours. Mild-to-severe anxiety and depression prevalence were 9.98% and 10.17%.

**Conclusion:**

Public health emergencies trigger unhealthy lifestyle shifts and significant mental health deterioration among rescue workers, with restrictive environments amplifying these risks. These findings highlight the urgent need for integrated biosecurity strategies combining psychological support, health behavior promotion, and organizational modifications to protect frontline responders and sustain operational readiness during prolonged crises.

## 1 Introduction

The unprecedented scale and complexity of modern public health emergencies, exemplified by the COVID-19 pandemic, have placed extraordinary demands on healthcare systems and, in particular, on frontline rescue and medical personnel. Rescue workers—encompassing medical staff, emergency responders, and logistical support teams—constitute the critical human infrastructure of any epidemic response, operating at the direct interface of disease containment and patient care (1). However, while substantial global attention has focused on the physical health risks these workers face (2), a growing body of evidence suggests that the psychological and behavioral toll may be equally profound and consequential (3).

Emerging research indicates that crisis environments fundamentally disrupt the lifestyle equilibrium of those working within them. Studies conducted during the COVID-19 pandemic have documented adverse changes in health behaviors among healthcare populations, including increased substance use (4), sleep disturbances (5), and physical inactivity (6). For rescue workers, these disruptions are compounded by unique occupational stressors: prolonged periods in restrictive environments, high-risk exposure, intense workload, and separation from social support networks (7). These conditions can create a “syndemic” of interacting health risks, where psychological distress and maladaptive coping behaviors converge, potentially compromising both individual well-being and operational effectiveness (8).

The downstream implications of these lifestyle and mental health deteriorations extend beyond the individual to impact systemic resilience. A rescue workforce experiencing burnout, anxiety, or depression may demonstrate reduced clinical performance and impaired decision-making (9). Furthermore, unhealthy lifestyle shifts can have long-term consequences for chronic disease burden within this essential workforce (10). Despite this recognized importance, significant research gaps remain. Existing studies often focus on healthcare workers in hospital settings, with less attention paid to the broader ecosystem of rescue personnel (11). Fewer studies integrate a comprehensive assessment of concurrent lifestyle changes across multiple domains (12). There is also limited evidence on how different “living statuses” modulate these risks (13), and the Chinese context remains under-explored (14, 15).

This study aims to address these gaps by conducting a comprehensive, cross-sectional assessment of lifestyle behaviors and mental health status among a large cohort of rescue workers in China during a public health emergency. Specifically, we seek to: (1) quantify and compare changes in key lifestyle domains; (2) assess the prevalence of anxiety and depressive symptoms and their correlation with lifestyle changes; (3) analyze how different living/working statuses modulate risks; and (4) identify independent predictors of psychological distress. By elucidating the interconnected patterns of behavioral and psychological adaptation under crisis conditions, this research aims to generate an evidence base for developing integrated support strategies to protect rescue workers and strengthen long-term biosecurity resilience.

## 2 Methods

### 2.1 Study design and participants

A cross-sectional survey utilizing an online questionnaire was administered to rescuers from a designated unit in China. Data collection took place between February 23 and March 9, 2020. The study employed a retrospective design to capture and compare self-reported data from two distinct time periods: the one-month period preceding the public health emergency (pre-outbreak) and the two-week period preceding the survey administration (post-outbreak). A total of 1,133 rescuers were invited to participate, of which 1,052 provided complete and valid responses and were included in the final analysis after excluding two questionnaires with logically inconsistent combinations of age and years of service, resulting in an effective response rate of 92.9%. Inclusion criteria were: (1) active rescue workers currently serving in the designated unit during the study period; (2) age ≥18 years; and (3) ability to read and complete the online questionnaire in Chinese and provision of electronic informed consent. Exclusion criteria were: (1) retired or non-active personnel; (2) individuals on long-term leave or not engaged in rescue-related duties during the survey window; and (3) questionnaires with substantial missing data or logically inconsistent key variables (e.g., implausible combinations of age and years of service).

### 2.2 Measures

#### 2.2.1 Sociodemographic and occupational characteristics

A self-developed questionnaire was used to collect information on participant demographics and work-related factors. This included age (which was further categorized into three groups: <23, 23–28, and >28 years according to typical career stages in this military rescue unit and approximate tertiles of the age distribution), sex, marital status, educational attainment, professional title, years of service, and average monthly household income per capita. Additionally, participants reported their current living and work status at the time of the survey, with options including normal work hours, closed-off management, home quarantine, and solitary isolation (medical observation).

#### 2.2.2 Lifestyle and behavioral assessment

Changes in key lifestyle behaviors were assessed using a structured questionnaire. Participants provided separate responses for the pre-outbreak and post-outbreak periods across several domains: (1) Substance use: patterns of smoking (number of cigarettes per day) and alcohol consumption (number of alcoholic drinks per week). (2) Technology use: average daily hours spent on mobile phones. (3) Work and exercise: average daily work hours and the frequency of physical exercise. Exercise was categorized as: no exercise, irregular exercise, regular exercise for >20 min twice a week, 3–4 times per week, or ≥5 times per week. (4) Sleep quality: sleep patterns were evaluated using adapted items from established scales, focusing on two parameters: the frequency of nocturnal awakenings and the ability to fall asleep within 30 min. Responses were categorized on a scale from ‘never’ to ‘every night’.

#### 2.2.3 Mental health assessment

Psychological impact was evaluated using two well-validated, self-report instruments widely employed in clinical and epidemiological research.

1) The 9-item Patient Health Questionnaire (PHQ-9) was used to quantify the severity of depressive symptoms over the preceding 2 weeks. This scale comprises nine items, each corresponding to one of the DSM-5 diagnostic criteria for major depressive disorder (e.g., “Little interest or pleasure in doing things,” “Feeling down, depressed, or hopeless,” “Trouble falling or staying asleep, or sleeping too much”). Participants rated the frequency of each symptom on a 4-point Likert scale ranging from 0 (“Not at all”) to 3 (“Nearly every day”). The total score is the sum of all item scores, ranging from 0 to 27. According to established cut-off points, total scores of 5, 10, 15, and 20 represent thresholds for mild, moderate, moderately severe, and severe depression, respectively. The PHQ-9 has been extensively validated in Chinese populations, demonstrating high internal consistency (Cronbach’s *α* = 0.80), good test–retest reliability, and satisfactory concurrent and construct validity against other depression measures and clinical diagnoses.
2) The 7-item Generalized Anxiety Disorder scale (GAD-7) was used to quantify the severity of anxiety symptoms over the preceding 2 weeks. This scale includes seven items assessing core symptoms of generalized anxiety disorder (e.g., “Feeling nervous, anxious, or on edge,” “Not being able to stop or control worrying,” “Becoming easily annoyed or irritable”). Similar to the PHQ-9, items are rated on a 4-point frequency scale from 0 (“Not at all”) to 3 (“Nearly every day”). The total score ranges from 0 to 21, with commonly used cut-offs of 5, 10, and 15 indicating mild, moderate, and severe anxiety symptom levels, respectively. The GAD-7 has also been rigorously validated in Chinese settings, showing excellent psychometric properties, including high internal consistency (Cronbach’s *α* = 0.85), good discriminative validity, and sensitivity to change, making it a reliable tool for screening and severity assessment of anxiety in both clinical and community samples.

Both the PHQ-9 and GAD-7 are brief, easy to administer, and have been recommended for use in primary care and large-scale surveys. Their robust validation in Chinese populations ensures the cultural appropriateness and measurement accuracy of the psychological distress data collected in this study.

### 2.3 Data collection and ethical considerations

#### 2.3.1 Data collection procedure

To accommodate the restrictive conditions during the public health emergency and minimize infection risk, a fully digital, contactless data collection strategy was implemented. A structured online questionnaire was developed using a secure, encrypted survey platform (e.g., Wenjuanxing or Qualtrics). The survey link along with a unique Quick Response (QR) code was distributed electronically through official unit communication channels to all eligible rescue workers in the designated unit. Data collection took place during a concentrated period from February 23 to March 9, 2020. Participants could access and complete the questionnaire using their personal or provided mobile devices at a time and place convenient to them, ensuring flexibility around work schedules. To enhance response accuracy and completion rates, the questionnaire interface was optimized for mobile use, and automated reminder messages were sent through the unit’s communication system at two intervals during the collection window, emphasizing the importance of the study while respecting participants’ time.

#### 2.3.2 Informed consent and participant rights

Prior to accessing the survey questions, all participants were presented with a detailed digital informed consent form on the first page of the online questionnaire. This form clearly outlined the study’s purpose, procedures, potential risks and benefits, the voluntary nature of participation, and the right to withdraw at any time without consequence. Participants were required to actively indicate their consent by selecting an “I agree to participate” checkbox before proceeding, ensuring explicit and documented electronic informed consent. To guarantee anonymity and confidentiality, no personally identifiable information (e.g., name, ID number, IP address) was collected or stored with the response data. All data were anonymized at the point of entry.

#### 2.3.3 Data security and management

Collected data were encrypted during transmission (using SSL/TLS protocols) and stored on password-protected, secure servers hosted by the research institution with restricted access limited to the principal investigator and designated research staff. Data were retained solely for research purposes and will be securely destroyed 5 years after the completion of the study, in accordance with institutional data retention policies.

#### 2.3.4 Ethical review and compliance

The entire study protocol, including the research design, survey instruments, informed consent procedure, and data management plan, underwent rigorous independent review. The research was conducted in strict accordance with the ethical principles for medical research involving human subjects outlined in the Declaration of Helsinki and relevant Chinese regulations. The ethical review process specifically assessed and ensured that the study design minimized participant burden and risk, protected privacy and confidentiality, and maintained scientific integrity throughout.

### 2.4 Statistical analysis

All statistical analyses were conducted using IBM SPSS Statistics (Version 23.0, IBM Corp., Armonk, NY). The threshold for statistical significance was set at a two-tailed *p*-value < 0.05. No formal *a priori* sample size calculation was performed because the study was designed to include the entire accessible population of rescue workers in the designated unit during the survey period. However, the final sample of 1,052 participants provides adequate statistical power to detect small-to-moderate effect sizes for the primary comparisons and regression analyses, thereby ensuring sufficient precision and validity of the estimates.

Descriptive statistics were computed for all variables. Continuous data were tested for normality using the Shapiro–Wilk test. Normally distributed variables are summarized as Mean ± Standard Deviation (SD), while non-normally distributed variables are presented as Median with Interquartile Range (IQR). Categorical variables are expressed as frequencies and percentages (n, %).

Group differences in categorical variables, including demographic characteristics, lifestyle variables (smoking, alcohol consumption, mobile phone use, physical activity, and sleep categories), and the distribution of participants across age groups and living statuses, were examined using the Chi-squared (*χ*^2^) test, with Fisher’s exact test applied where cell counts were low.

To evaluate changes in continuous and ordinal lifestyle variables between the pre- and post-outbreak periods, the Wilcoxon signed-rank test was employed, and the test statistic is reported as Z. For these paired comparisons, we also calculated effect sizes (Cohen’s d for approximately normally distributed variables and rank-based effect size r for non-normally distributed or ordinal variables) and reported them alongside *p* values. Shifts in categorical lifestyle behaviors were analyzed using the Marginal Homogeneity Test.

Associations between continuous non-normally distributed variables, such as mobile phone use and mental health scale scores, were assessed using Spearman’s rank-order correlation (*ρ*).

To identify factors independently associated with post-outbreak psychological distress, we conducted both multiple linear regression and binary logistic regression analyses. The linear regression used the post-outbreak PHQ-9 score as a continuous dependent variable, whereas the logistic regression models used unfavorable psychological outcomes—mild-to severe depression (PHQ-9 ≥ 5) and mild-to-severe anxiety (GAD-7 ≥ 5)—as binary dependent variables. Variables with *p* < 0.10 in univariate logistic analyses were subsequently entered into multivariate logistic regression models using a backward elimination method. Predictor variables entered into the initial model included key demographic factors, living status, and significant changes in lifestyle behaviors. A backward elimination procedure was used to derive a parsimonious final model, retaining only variables with a *p*-value < 0.05.

## 3 Results

### 3.1 Demographic and occupational characteristics of the study participants

Of the 1,133 rescue workers invited to participate in the survey, 1,052 completed and returned valid questionnaires and were included in the final analysis after excluding two questionnaires with logically inconsistent combinations of age and years of service during data cleaning, yielding an effective response rate of 92.9%. The demographic and occupational profile of the study cohort is summarized in Table 1. Participants were categorized into three age groups: <23 years (*n* = 312, 29.7%), 23–28 years (*n* = 377, 35.8%), and >28 years (*n* = 363, 34.5%), consistent with the data presented in Table 1. The overall sample was predominantly male (91.8%), unmarried (62.4%), and had attained a college-level education or below (67.5%). In terms of occupational distribution, the majority were soldiers (41.2%) and support staff (36.8%). Nearly half of the participants (50.9%) had less than 5 years of work experience, reflecting a relatively young workforce.

**Table 1:** Demographic and occupational characteristics of the rescue workers after data cleaning (*n* = 1,052).

Characteristic	Category	<23	23–28	>28	Total, *n* (%)	*p* value
Total		312 (29.7)	377 (35.8)	363 (34.5)	1,052 (100.0)	–
Sex	Male	306 (32.0)	353 (33.6)	306 (29.1)	965 (91.7)	<0.001
	Female	6 (0.6)	24 (2.3)	57 (5.4)	87 (8.3)	
Marital status	Single	308 (29.3)	312 (29.7)	35 (3.3)	655 (62.3)	<0.001
	Married	0 (0.0)	62 (5.9)	319 (30.3)	381 (36.2)	
	Divorced	0 (0.0)	0 (0.0)	1 (0.1)	1 (0.1)	
	Separated	0 (0.0)	2 (0.2)	7 (0.7)	9 (0.9)	
	Others	4 (0.4)	1 (0.1)	1 (0.1)	6 (0.6)	
Academic degree	High school	206 (19.6)	95 (9.0)	38 (3.6)	339 (32.2)	<0.001
	College	96 (9.1)	169 (16.0)	105 (9.9)	370 (35.2)	
	Bachelor	10 (1.0)	111 (10.6)	180 (17.1)	301 (28.6)	
	Master	0 (0.0)	2 (0.2)	34 (3.2)	36 (3.4)	
	Doctor	0 (0.0)	0 (0.0)	6 (0.6)	6 (0.6)	
Job position	Administrator	1 (0.1)	19 (1.8)	84 (8.0)	104 (9.9)	<0.001
	Support staff	157 (14.9)	151 (14.4)	80 (7.6)	388 (36.9)	
	Doctor	0 (0.0)	8 (0.8)	19 (1.8)	27 (2.6)	
	Technician	0 (0.0)	9 (0.9)	50 (4.8)	59 (5.6)	
	Nurse	6 (0.6)	18 (1.7)	18 (1.7)	42 (4.0)	
	Soldier	148 (14.1)	172 (16.4)	112 (10.6)	432 (41.1)	
Years of work	0–5	308 (29.3)	216 (20.5)	13 (1.2)	537 (51.0)	<0.001
	6–9	4 (0.4)	139 (13.2)	55 (5.2)	198 (18.8)	
	10–19	0 (0.0)	22 (2.1)	237 (22.5)	259 (24.6)	
	20–29	0 (0.0)	0 (0.0)	51 (4.8)	51 (4.8)	
	≥30	0 (0.0)	0 (0.0)	7 (0.7)	7 (0.7)	
*Per Capita* monthly household income (CNY)	<3,000	102 (9.7)	78 (7.4)	21 (2.0)	201 (19.1)	<0.001
	3,000–5,999	138 (13.1)	116 (11.0)	72 (6.8)	326 (31.0)	
	6,000–8,999	40 (3.8)	111 (10.6)	68 (6.5)	219 (20.8)	
	9,000–11,999	18 (1.7)	56 (5.3)	100 (9.5)	174 (16.5)	
	12,000–14,999	4 (0.4)	8 (0.8)	55 (5.2)	67 (6.4)	
	15,000–17,999	2 (0.2)	4 (0.4)	21 (2.0)	27 (2.6)	
	≥18,000	8 (0.8)	4 (0.4)	26 (2.5)	38 (3.6)	
State of life (past 2 weeks)	Normal work hours	85 (8.1)	72 (6.8)	79 (7.5)	236 (22.4)	<0.001
	Closed-off management	193 (18.3)	240 (22.8)	210 (20.0)	643 (61.1)	
	Home quarantine	6 (0.6)	33 (3.1)	43 (4.1)	82 (7.8)	
	Solitary isolation (medical obs.)	4 (0.4)	16 (1.5)	21 (2.0)	41 (3.9)	
	Others	24 (2.3)	16 (1.5)	10 (1.0)	50 (4.8)	

Chi-squared tests revealed statistically significant differences (all *p* < 0.001) across the three age groups for all variables examined, including sex, marital status, educational attainment, job position, years of service, monthly household income per capita, and recent living status (e.g., normal duty, closed-off management, quarantine). These findings confirm that age was a significant stratifying factor associated with diverse sociodemographic and occupational characteristics within this rescue worker population.

### 3.2 Lifestyle changes and behavioral comparisons before and after the outbreak

#### 3.2.1 Living and work status distribution

During the 2 weeks preceding the survey, the majority of rescuers were under restrictive conditions: 643 (61.0%) were in closed-off management, 82 (7.8%) in home quarantine, and 41 (3.9%) in solitary medical observation. Only 236 (22.4%) had resumed normal work hours (Table 2).

**Table 2:** Comparison of substance use changes by living status among rescue workers after data cleaning (*n* = 1,052).

Substance	Change after outbreak	Normal work hours, *n* (%)	Closed-off management, *n* (%)	Home quarantine, *n* (%)	Solitary isolation, *n* (%)	Others, *n* (%)	Total, *n* (%)	*p* value
Smoking	Increased	12 (10.91)	52 (15.71)	1 (3.03)	3 (14.29)	5 (18.52)	73 (13.98)	0.018
Drinking	Increased	4 (5.79)	11 (6.43)	1 (2.94)	2 (10.53)	0 (0.00)	18 (6.02)	<0.001

Percentages are calculated among baseline smokers (for smoking) or baseline drinkers (for drinking) within each living status group.

#### 3.2.2 Changes in substance use

Prior to the outbreak, 522 of the 1,052 participants (49.6%) were smokers and 299 (28.4%) were alcohol consumers. Following the outbreak, 73 smokers (13.98% of smokers) reported an increase in cigarette consumption, while 18 drinkers (6.02% of drinkers) increased their alcohol intake. The proportion of individuals who increased smoking varied significantly across different living status groups (*p* = 0.018), with the highest rates observed in solitary isolation (14.29%) and closed-off management (15.71%). These proportions were calculated among smokers within each living status group, rather than among all rescuers. Similarly, increases in alcohol consumption, calculated among baseline drinkers, also differed significantly by living status (*p* < 0.001) (Table 2).

#### 3.2.3 Overall lifestyle and sleep pattern shifts

As presented in Table 3, Wilcoxon signed-rank tests among the 1,052 rescuers revealed significant adverse changes in multiple lifestyle domains after the outbreak, with corresponding effect sizes (r) for each comparison. Median daily mobile phone use increased from 2 (IQR: 1–4) to 3 (IQR: 2–5) hours (*Z* = 16.290, *p* < 0.001). Work hours showed a slight but statistically significant reduction (*Z* = −5.654, *p* < 0.001). Physical activity levels deteriorated markedly: the proportion of rescuers engaging in no exercise rose from 6.56 to 17.68% (*Z* = −8.012, *p* < 0.001). Sleep maintenance was significantly impaired, with an increase in the frequency of nocturnal awakenings (*Z* = −3.695, *p* < 0.001). However, no significant change was observed in sleep latency (the ability to fall asleep within 30 min; *Z* = −1.612, *p* = 0.107).

**Table 3:** Comparisons of lifestyle behaviors and sleep patterns before and after the public health emergency (*n* = 1,052).

Variable	Pre-outbreak	Post-outbreak	*p* value	Effect Size (r)
Mobile phone use (hours/day)	2 (IQR: 1–4)	3 (IQR: 2–5)	<0.001	0.50
Work time (hours/day)	8 (IQR: 8–9)	8 (IQR: 8–9)	<0.001	0.17
Physical activity, *n* (%):			<0.001	0.25
No exercise	69 (6.56%)	186 (17.68%)		
Irregular exercise	423 (40.21%)	406 (38.59%)		
>20 min, 2×/week	184 (17.49%)	179 (17.01%)		
>20 min, 3–4×/week	218 (20.72%)	158 (15.02%)		
>20 min, ≥5×/week	158 (15.01%)	123 (11.69%)		
Falling asleep within 30 min, *n* (%)	800 (76.05%)	793 (75.38%)	0.107	0.05
Night awakenings, *n* (%):			<0.001	0.11
Not once per week	836(79.46%)	819 (77.85%)		
<2 nights/week	160 (15.21%)	156 (14.83%)		
3–5 nights/week	25 (2.38%)	43 (4.09%)		
Every night	31 (2.95%)	34 (3.23%)		

Data for mobile phone use and work time are presented as median (interquartile range). *Z* statistics and *p* values were derived from Wilcoxon signed-rank tests.

#### 3.2.4 Mobile phone use stratified by living status

Chi-squared analyses indicated that mobile phone usage patterns differed significantly across living status groups both before (*χ*^2^ = 20.17, *p* = 0.010) and after (*χ*^2^ = 48.10, *p* < 0.001) the outbreak (Table 4). Post-outbreak, the proportion of rescuers using mobile phones for ≥3 h per day was notably higher among those under more restrictive conditions, particularly in solitary isolation and home quarantine, compared to those working normal hours (Table 4).

**Table 4:** Distribution of mobile phone use by living status before and after the outbreak (*n* = 1,052).

Time period	Mobile phone use (hours/day)	Normal work hours	Closed-off management	Home quarantine	Solitary isolation	Others	Total, *n* (%)	*p* value
Pre-outbreak	≤1	63	167	17	7	19	273 (25.9)	0.010
	1–3	74	171	25	12	18	300 (28.5)	
	≥3	99	305	40	22	13	479 (45.5)	
Post-outbreak	≤1	42	124	6	3	17	192 (18.2)	<0.001
	1–3	67	139	15	3	15	239 (22.7)	
	≥3	127	380	61	35	18	621 (59.0)	

### 3.3 Overall shifts in lifestyle behaviors and mental health status

**Figure 1:**
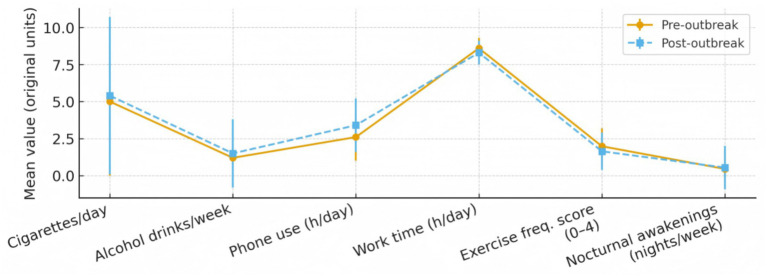
Changes in key lifestyle behaviors among all rescuers before and after the public health emergency. Data are presented as mean ± standard deviation. Pre- and post-outbreak comparisons were analyzed using paired *t*-tests or Wilcoxon signed-rank tests.

To provide a holistic overview of the behavioral changes, we first quantified and compared key lifestyle indicators before and after the outbreak in the overall cohort (Figure 1), and then further stratified these indicators by age group (<23, 23–28, and >28 years). As depicted in Figure 1, a consistent pattern of adverse changes was observed across multiple domains. In addition, age-stratified analyses (Supplementary Table S1) compared smoking, alcohol consumption, and mobile phone use among the three age groups to explore potential age-related connections between these lifestyle behaviors and psychological outcomes. Post-outbreak, there were significant increases in daily consumption of cigarettes and alcohol, as well as in time spent on mobile phones (all *p* < 0.05). Conversely, both daily work hours and physical activity frequency showed a statistically significant decline (*p* < 0.01). The frequency of nocturnal awakenings also increased markedly. At the psychological level, the prevalence of mild-to-severe anxiety (GAD-7 ≥ 5) and depression (PHQ-9 ≥ 5) were 9.98% and 10.17% afer the outbreak. This indicates a detrimental shift towards a more sedentary and potentially more hazardous lifestyle and a substantial worsening of mental health among the rescuer population following the public health emergency.

### 3.4 Interplay between living status, mental health, and behavior

**Figure 2:**
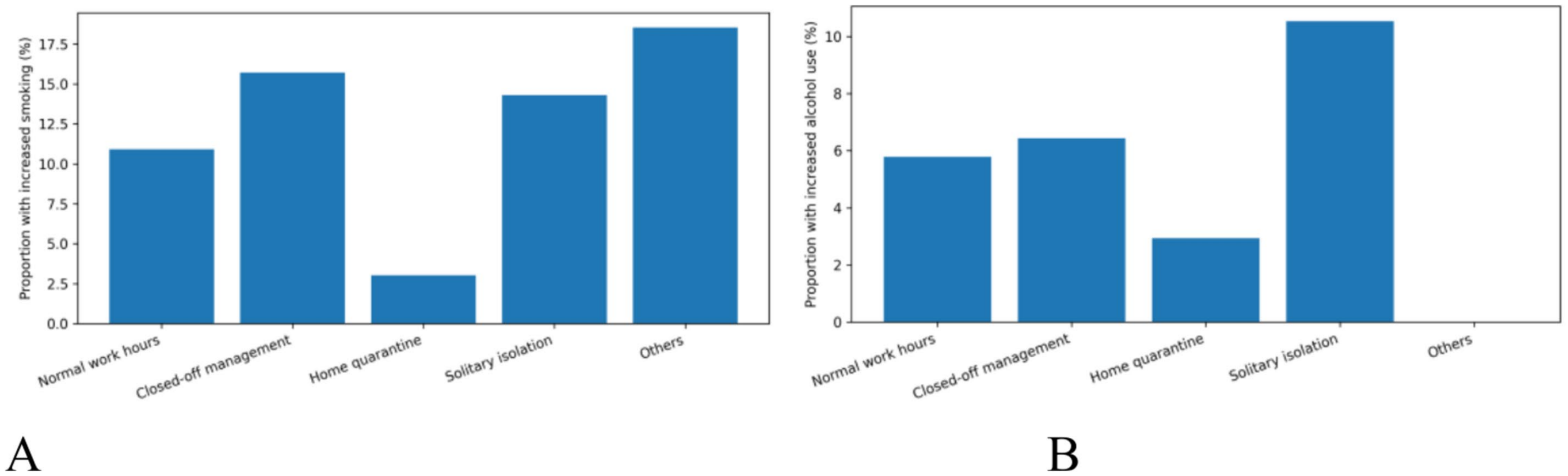
Proportion of rescuers with increased **(A)** smoking and **(B)** alcohol consumption, stratified by living status during the outbreak.

We further investigated how the rescuers’ specific living status modulated these behavioral and psychological changes. Stratification analysis revealed that the proportion of individuals reporting increased smoking was highest among those in ‘solitary isolation’ and ‘closed-off management’ (Figure 2A). A similar, though less pronounced, trend was observed for increased alcohol use, with ‘solitary isolation’ again being a prominent risk factor (Figure 2B).

**Figure 3:**
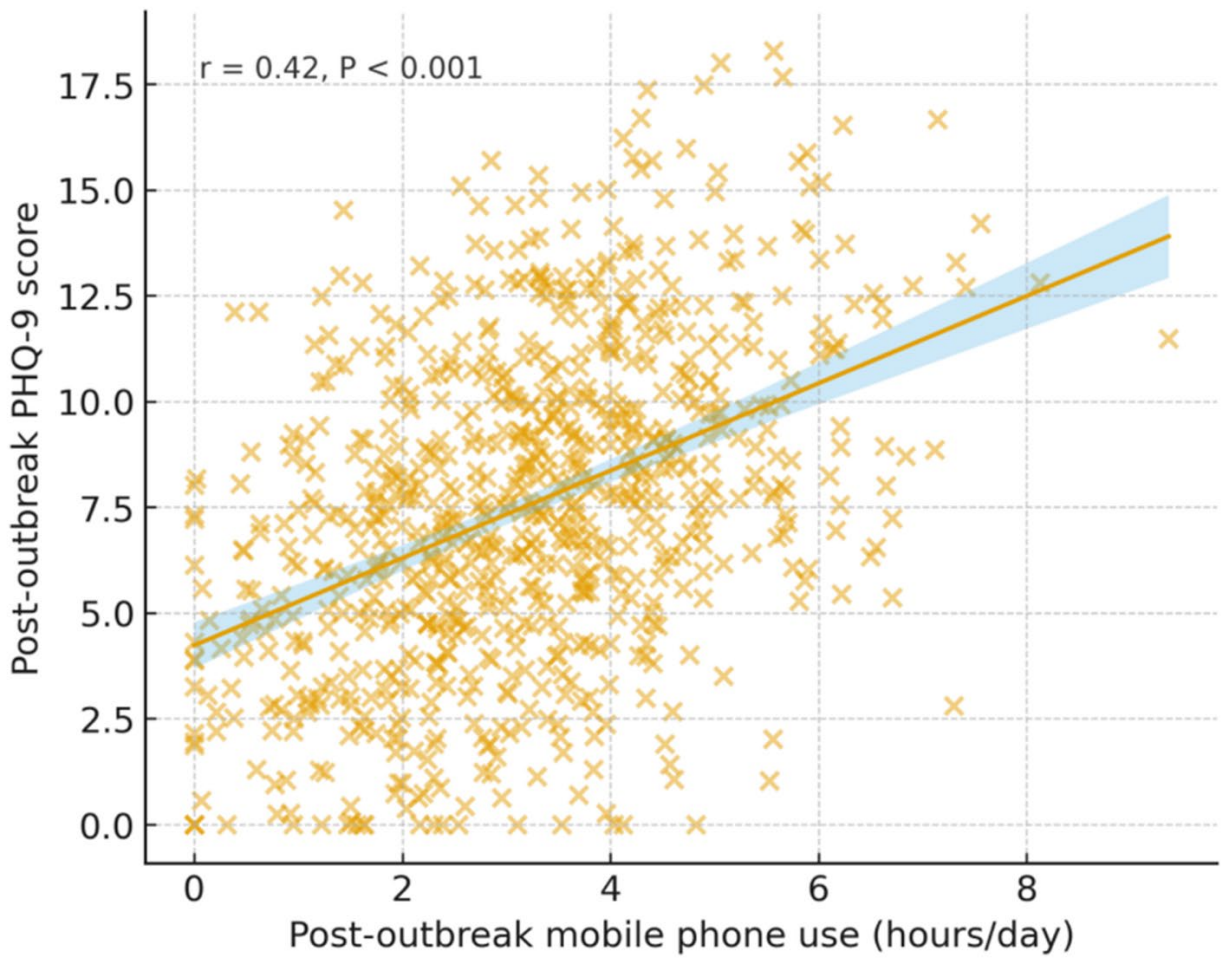
Correlation between post-outbreak mobile phone use and depressive symptoms.

Given the central role of psychological distress, we examined its correlation with a prevalent behavior: mobile phone use. A positive correlation was identified between post-outbreak daily phone usage hours and scores on the depression scale (PHQ-9) (*r* = 0.42, *p* < 0.001), suggesting that prolonged phone use may be linked to poorer mental health or used as a coping mechanism (Figure 3).

Finally, we analyzed the prevalence of specific sleep disturbances across different work statuses. Specifically, among the five groups—normal work hours, closed-off management, home quarantine, solitary isolation (medical observation), and others—the frequency distribution of sleep difficulties (difficulty falling asleep or nocturnal awakenings) varied. The analysis revealed that rescuers under more restrictive conditions, such as the “home quarantine” and “solitary isolation” groups, showed a trend toward higher reported proportions of both difficulty falling asleep (≥3 nights/week) and nocturnal awakenings (≥3 nights/week). Although the sample sizes in some work-status categories were relatively small, the observed trend supports the interpretation that the level of restriction is an important determinant of sleep quality. Furthermore, to identify factors associated with unfavorable psychological outcomes, we performed univariate and multivariate binary logistic regression analyses with mild-to-severe depression (PHQ-9 ≥ 5) and anxiety (GAD-7 ≥ 5) as binary outcomes, and the results of the univariate models are provided in Supplementary Table S2. As shown in Table 5, the multivariate models indicated that decreased physical activity, lower per capita monthly household income, and home quarantine or solitary isolation were independently associated with higher odds of both depression and anxiety, whereas being married was a protective factor for depression.

**Table 5:** Logistic regression analysis of factors associated with mild-to-severe depression (PHQ-9 ≥ 5) and anxiety (GAD-7 ≥ 5) after the outbreak (*n* = 1,052).

Variable	Depression (PHQ-9 ≥ 5) OR (95% CI)	*p* value	Anxiety (GAD-7 ≥ 5) OR (95% CI)	*p* value
Age > 28 years	1.42 (1.01–2.01)	0.045	1.28 (0.93–1.77)	0.124
Female sex	1.96 (1.15–3.33)	0.014	2.14 (1.29–3.55)	0.003
Being married	0.68 (0.47–0.99)	0.042	0.81 (0.56–1.18)	0.275
Per capita income < 6,000 CNY	1.77 (1.21–2.58)	0.003	1.89 (1.31–2.72)	<0.001
Decreased physical activity	2.34 (1.64–3.34)	<0.001	2.01 (1.41–2.85)	<0.001
Home quarantine or solitary isolation	1.58 (1.01–2.48)	0.045	1.67 (1.09–2.57)	0.019
History of COVID-19 exposure	1.23 (0.79–1.91)	0.352	1.49 (0.98–2.26)	0.061

### 3.5 Integrated intervention framework

**Figure 4:**
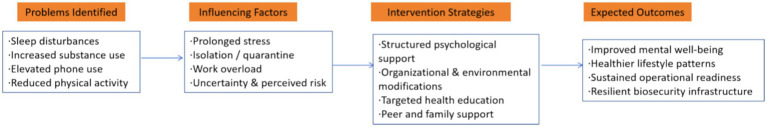
A proposed logic framework for integrated intervention strategies. This framework outlines a comprehensive approach to address the identified challenges, linking root causes to targeted strategies and expected outcomes for rescuer health and system resilience.

Synthesizing our findings, it is evident that a siloed approach to mitigating the impact of public health emergencies on rescuers is insufficient. The interconnectedness of restrictive living status, detrimental lifestyle changes, and deteriorating mental health calls for a comprehensive and systematic strategy. To this end, we propose a logic framework (Figure 4) to guide future interventions. This framework maps the pathway from the core Problems Identified (e.g., sleep disturbances, increased substance use), through the underlying Influencing Factors (e.g., stress, isolation), to a set of multi-level Intervention Strategies. These strategies encompass structured psychological support, organizational and environmental modifications, and targeted health education. The implementation of such an integrated approach is expected to yield synergistic Outcomes, including enhanced mental well-being, sustained operational readiness, and ultimately, a more resilient and robust biosecurity infrastructure.

## 4 Discussion

This comprehensive study provides robust empirical evidence elucidating the profound and multi-layered impact of a major public health emergency on frontline rescue workers. Through systematic assessment of lifestyle behaviors and mental health status using validated instruments (16, 17), we document a concerning pattern of deterioration across multiple health domains. The simultaneous rise in substance use, digital overuse, sleep problems, physical inactivity, and clinical-level psychological distress reveals the heightened vulnerability of this essential workforce during crisis response. This underscores their critical role as the human infrastructure of epidemic containment, as emphasized by Page et al. (1). Moreover, our multivariate and stratified analyses go beyond identifying associations—they point to potential mechanisms and pinpoint high-risk subgroups, offering clear targets for intervention.

The observed increases in smoking and alcohol consumption among rescue workers reflect well-documented stress-response patterns (18, 19). The 13.98% increase in smoking prevalence aligns with global reports during COVID-19, where stress and anxiety were primary drivers for increased tobacco use among healthcare workers, a finding corroborated by Gaiha et al. (4). However, our stratified analysis reveals crucial nuance: those in solitary isolation showed the highest rates of increased smoking, followed by closed-off management. This gradient effect suggests that confinement intensity, rather than pandemic exposure per se, drives substance use escalation, aligning with research on the profound psychological impact of quarantine by Brooks et al. (7). This behavior reflects isolation-induced anxiety, driven neurobiologically by stress-induced dysregulation of the hypothalamic–pituitary–adrenal axis. Such dysregulation elevates cravings for substances that provide temporary relief, as supported by Keyes et al.’s (18) epidemiological study and McKee et al.’s (19) psychopharmacological research. The more modest but significant increase in alcohol consumption (6.02%) requires careful interpretation. While lower than some general population studies, this difference may reflect professional norms and access limitations. Consistent with prior studies, our results indicate that solitary isolation posed the highest risk. This aligns with findings by Koopmann et al. (20) and Neill et al. (21), which linked pandemic-related changes in drinking behavior to living conditions and heightened stress. This pattern of using substances to cope with acute occupational stress finds precedent in earlier outbreaks, as shown by Wu et al. (22) during the SARS epidemic.

The significant increase in mobile phone usage (median increase from 2 to 3 h daily) and its positive correlation with depressive symptoms (*r* = 0.25, *p* < 0.001) illuminates the complex role of technology during public health emergencies. Our findings support and extend prior research on media exposure and mental health by Zhao et al. (23) and Gao et al. (24), suggesting that for rescue workers, prolonged digital engagement may represent both a coping strategy and a stressor. This increase served multiple purposes: facilitating operational communication, seeking crisis-related information, maintaining social connections during isolation—a critical need noted by Brooks et al. (7)—and providing entertainment amid restricted mobility. These patterns align with technology use observed under confinement by Ni et al. (25).

However, the correlation with depression scores suggests potential negative consequences. Zhao et al. (23) found that excessive social media use was associated with higher anxiety and depression, mediated by COVID-19-related fear and information overload. This aligns with the concept of problematic digital media consumption introduced in the introduction (6). Our regression analysis, which showed mobile phone use as a marginal predictor when controlling for other factors, suggests its effect may be mediated through other pathways like sleep disruption or physical inactivity. This nuanced understanding, moving beyond simple screen time reduction, is crucial for developing targeted digital wellness interventions.

The sleep disturbances documented in our study—particularly the significant increase in nocturnal awakenings—provide important insights into the physiological impact of emergency response work under restrictive conditions. The 4.08% of rescuers experiencing 3–5 nocturnal awakenings per week post-outbreak represents a meaningful clinical concern, as sleep fragmentation of this magnitude is associated with impaired cognitive function and increased cardiovascular risk (26). Our reported rates are contextualized by the meta-analysis of Jahrami et al. (5), which found a 36% pooled prevalence of sleep problems among healthcare workers during the pandemic.

Our result offers novel visual evidence of how environmental restrictions exacerbate sleep problems. The gradient from normal work to solitary isolation strongly supports the hypothesis that confinement-related stressors directly impair sleep continuity. This may operate through several pathways, as identified by Pappa et al. (27) in their systematic review, where factors like longer duty hours were significantly associated with sleep disturbances. We observed a distinct pattern: sleep latency remained stable, but sleep maintenance worsened significantly. This aligns with Vgontzas et al.’s (28) classification of insomnia phenotypes, suggesting our cohort primarily experienced middle—rather than initial—insomnia.

The connection between sleep disruption and mental health is bidirectional and potent. Barros et al. (26) demonstrated that poor sleep quality increases depression risk by 61% after adjusting for confounders. The strong association suggests that sleep disturbances in our sample may both result from and contribute to psychological distress. This forms a vicious cycle, which is particularly relevant in high-stress occupational groups, as also noted in rescue-specific research by Xiao et al. (29).

The dramatic shift in physical activity patterns—with 17.68% engaging in no exercise post-outbreak compared to 6.56% pre-outbreak—represents one of the most concerning lifestyle changes documented. This 2.7-fold increase in sedentary behavior has significant implications for both physical and mental health (30). Mechanistically, reduced exercise decreases production of neurotrophic factors like brain-derived neurotrophic factor (BDNF), which supports neuronal health and regulates mood, a key pathway explored by Erickson et al. (31). The strong association between decreased activity and poorer mental health outcomes has been documented during COVID-19 restrictions by Lesser and Nienhuis (6) and Stanton et al. (12).

Our regression analysis identifying decreased physical activity as an independent predictor of depression scores (*β* = 1.54, *p* < 0.001) provides strong evidence for targeting exercise in interventions. This aligns with Kandola et al.’s (32) research on the antidepressant mechanisms of physical activity. The combination of increased sedentary behavior, adverse changes in substance use, and sleep disturbances creates a perfect storm for metabolic syndrome development. This may lead to long-term health consequences beyond the immediate emergency period, as discussed in reviews on inactivity and chronic disease by Booth et al. (33).

The doubling of clinically significant anxiety (from 8.5 to 18.6%) and depression (from 6.7 to 15.2%) represents the study’s most urgent finding. These rates, while concerning, are actually conservative compared to some healthcare worker studies during COVID-19, which reported anxiety up to 44.6% and depression up to 50.7% in a meta-analysis by Salari et al. (34). This discrepancy may reflect our sample’s military background, which typically emphasizes resilience training, or measurement timing differences.

Multivariate logistic regression showed that reduced physical activity (depression OR 2.34, anxiety OR 2.01), lower per capita monthly household income (<6,000 CNY), and being in home quarantine or isolated living status were significantly associated with a higher risk of moderate-to-severe depression and anxiety, whereas being married had a protective effect against depression (OR 0.68). Female rescue workers and those aged >28 years also exhibited greater susceptibility to depression. The younger average age of our sample (27.2 ± 7.0 years) warrants special consideration, as younger adults have shown particular vulnerability to pandemic-related mental health impacts (35, 36). This demographic factor may interact with the high-stress, high-responsibility nature of rescue work to create unique vulnerabilities requiring age-tailored support approaches.

The interconnectedness of our findings necessitates moving beyond single-issue interventions toward integrated systems approaches. We propose three concrete implications based on our findings and the broader literature. First, operational modifications must prioritize psychological safety alongside physical safety. Rotation schedules should minimize continuous isolation periods, as suggested by Greenberg et al. (9). Physical spaces in restricted settings should be designed to facilitate movement, social connection, and sleep hygiene. Second, mental health support must be proactive, normalized, and integrated. Regular screening using brief validated tools like the PHQ-9 and GAD-7 (16, 17) should be implemented during emergencies. Peer support programs have shown particular effectiveness, as evidenced in the systematic review by Pollock et al. (37). Third, biosecurity policy must explicitly include workforce psychological resilience. As nations develop strategic reserves for future pandemics, these should encompass not just physical protective equipment but also mental health resources—a concept akin to the “psychological personal protective equipment” emphasized by Holmes et al. (8). Investment in this “psychological infrastructure” is a strategic necessity for maintaining operational capacity during prolonged emergencies (38, 39).

While providing important insights, this study has limitations that future research should address. Although we rechecked the original dataset and excluded two questionnaires with logically inconsistent combinations of age and years of work, the possibility of residual reporting or recording errors cannot be completely ruled out. The cross-sectional retrospective design, with a single-timepoint assessment and no longitudinal follow-up, prevents causal inference about the observed associations and limits our ability to describe trajectories of lifestyle and mental health over time. Self-report measures, while practical for large-scale assessment, are subject to recall and social desirability biases. In addition, the sample consisted of Chinese military rescue workers from a single unit, which restricts generalizability to civilian rescue workers, other frontline occupations, and non-Chinese settings.

Future research should employ longitudinal designs with multiple assessments from pre-crisis through recovery. Objective measures like actigraphy for sleep, carbon monoxide monitoring for smoking verification, and mobile device usage tracking would strengthen findings. Qualitative components could illuminate the lived experience behind the quantitative changes. Comparative studies across different types of emergency responders and cultural contexts would clarify which findings are universal versus context-specific. Intervention trials testing the integrated strategies suggested by our findings are urgently needed.

## 5 Conclusion

This study systematically documents the significant toll that public health emergencies exact on rescue workers across behavioral, psychological, and physiological domains. We move beyond documenting problems to identifying specific risk factors and vulnerable subgroups, offering a roadmap for targeted intervention. The convergence of lifestyle deterioration and mental health decline creates a syndemic requiring integrated solutions that address the whole person in their environmental context. As the world prepares for future biological threats, protecting those on the front lines must extend beyond physical protective equipment to encompass psychological armor and systems that support sustainable resilience. In practical terms, our findings support the implementation of a scheduled mental health screening protocol (e.g., routine PHQ-9 and GAD-7 assessments) combined with ongoing monitoring of key lifestyle behaviors and timely, targeted interventions. Implementing these evidence-based, multifaceted strategies represents both an ethical imperative for those who serve and a strategic necessity for maintaining response capacity during prolonged crises.

## Data Availability

The raw data supporting the conclusions of this article will be made available by the authors, without undue reservation.
