# Supplementary material for: Lifestyle consequences for rescue workers in public health emergencies: a cross-sectional study from china
Source: Front Public Health. 2026 Feb 27;14:1758053. doi: 10.3389/fpubh.2026.1758053 (PMC12982439; doi:10.3389/fpubh.2026.1758053)
Supplement: Supplementary file 1 [file Data_Sheet_1.pdf]

Supplementary Table S1.

Lifestyle Variables by Age Group among Participants with Mild-to-Severe Depression or Anxiety

Variable	<23 years n (%)	23–28 years n (%)	>28 years n (%)	*P* value
Smoking (Yes, among affected)	15 (4.8%)	38 (10.1%)	55 (15.2%)	<0.001
Alcohol Consumption (Yes, among affected)	28 (8.9%)	45 (11.9%)	67 (18.5%)	0.004
Daily Mobile Use >4h	205 (65.3%)	230 (60.8%)	184 (50.8%)	0.002
Decreased Physical Activity	191 (60.8%)	218 (57.7%)	175 (48.3%)	<0.001
Poor Sleep Quality	172 (54.8%)	206 (54.5%)	152 (42.0%)	<0.001

Note: Subgroup includes participants with PHQ-9 or GAD-7 ≥5 not representative of full sample smoking or alcohol use prevalence.

Supplementary Table S2.

Results of Univariate Logistic Regression Analyses for Factors Associated with Depression and Anxiety Among Rescue Workers

**Variable**	**B (Depression)**	**SE (Depression)**	**Wald (Depression)**	**OR (95% CI) (Depression)**	***P* value (Depression)**	**B (Anxiety)**	**SE (Anxiety)**	**Wald (Anxiety)**	**OR (95% CI) (Anxiety)**	***P* value (Anxiety)**
Sex (Female vs Male)	0.85	0.22	14.8	2.34 (1.52–3.59)	<0.001	0.9	0.23	15.3	2.47 (1.57–3.88)	<0.001
Marital status (Married vs Others)	-0.87	0.2	18.9	0.42 (0.28–0.63)	<0.001	-0.58	0.21	7.7	0.56 (0.37–0.84)	0.005
Academic degree (≥Bachelor vs <Bachelor)	-0.42	0.16	6.6	0.66 (0.49–0.89)	0.007	-0.3	0.16	3.4	0.74 (0.54–1.02)	0.063
Job position (Medical staff vs Others)	0.68	0.29	5.5	1.98 (1.12–3.50)	0.019	0.77	0.3	6.7	2.16 (1.20–3.88)	0.010
Years of work (≥10 vs <10)	0.45	0.17	7	1.57 (1.12–2.21)	0.009	0.48	0.18	7.2	1.61 (1.13–2.30)	0.008
Income (≥9000 vs <9000)	-0.97	0.2	23.5	0.38 (0.26–0.56)	<0.001	-0.8	0.21	14.8	0.45 (0.30–0.68)	<0.001
Living status (Quarantine/Isolation vs Normal)	0.96	0.17	31.9	2.61 (1.88–3.63)	<0.001	0.89	0.17	27.2	2.44 (1.74–3.42)	<0.001
Physical activity (Decreased vs Not decreased)	1.17	0.15	59.3	3.21 (2.38–4.32)	<0.001	1.12	0.15	56	3.07 (2.26–4.17)	<0.001

Note: Logistic regression models were conducted separately for depression (PHQ-9 ≥5) and anxiety (GAD-7 ≥5). B = regression coefficient; SE = standard error;

OR = odds ratio; CI = confidence interval. Significant associations ($P < 0.05$) are indicated in bold.
